# Australian neonatal nurses’ professional quality of life: A descriptive cross-sectional study

**DOI:** 10.1177/13674935251339351

**Published:** 2025-05-02

**Authors:** Patricia L. Lowe, Samantha Jakimowicz, Tracy L. Levett-Jones, Daniel Demant

**Affiliations:** 1 School of Nursing and Midwifery, 110561 University of Technology Sydney, Sydney, NSW, Australia; 2 School of Nursing, Midwifery and Paramedicine, 89470 Charles Sturt University, Bathurst, NSW, Australia; 3 Course Director (Bachelor of Health Science), School of Public Health, Faculty of Health, 110561 University of Technology Sydney, Sydney, NSW, Australia

**Keywords:** Burnout, psychological, compassion fatigue, nurses, neonatal, quality of life

## Abstract

This descriptive cross-sectional study investigated the demographic profile of surveyed Australian neonatal nurses, explored their self-reported professional quality of life status, and appraised the strength and direction of relationships between these variables. Australian College of Neonatal Nurses (ACNN) general members (*N* = 950) were invited to participate. An online Qualtrics^TM^ survey was distributed via email. Compassion satisfaction (CS) and fatigue (CF) scores were measured using the Modified Professional Quality of Life Scale (ProQOL-21]). Spearman’s correlation calculated the strength and direction of relationships between variables. Fifty-three neonatal nurses responded to the survey (*N* = 950, response rate = 5.58%). Respondents reported moderate to high-level compassion satisfaction and high-level compassion fatigue. Nurses in clinical roles revealed higher compassion fatigue scores than those in non-clinical roles. A statistically significant positive correlation was detected between years of experience in neonatal care and compassion satisfaction (r = 0.277, 95% CI [0.002, 0.513]). These findings question the belief that education and experience safeguard against work-related stress and emphasise that strategies to offset the fatigue reported by this female-dominated workforce are needed.

## Introduction

Research examining nurses’ and midwives’ varying demographic profiles and experience levels emphasises professional quality of life’s complex and multi-faceted nature (Barr, 2022). Professional quality of life (Pro-QOL) is defined as ‘the quality one feels in relation to their work as a helper’ (Stamm, 2010: p. 8). A healthcare professional’s quality of life reflects the perceived cumulative impact of work-based stress (compassion fatigue) and satisfaction (compassion satisfaction) on their physical and psychological health (Stamm, 2010). Compassion satisfaction (CS) reflects ‘the pleasure, purpose, and gratification received by professional caregivers through their contributions to the well-being of patients and their families’ (Sacco and Copel, 2017: p.78). Conversely, compassion fatigue (CF) is defined as:

‘A preventable state of holistic exhaustion that manifests as a physical decline in energy and endurance, an emotional decline in empathetic ability and emotional exhaustion, and a spiritual decline as one feels hopeless or helpless to recover that results from chronic exposure to others’ suffering, compassion, high-stress exposure, and high occupational use of self in the absence of boundary setting and self-care measures’ (Peters, 2018: p. 470).

A recently conducted critical appraisal of internationally published nursing and midwifery literature compared how CS and CF affect nurses and midwives practicing in geographically diverse clinical settings and at all stages of their careers (Zhang et al., 2018). It was found that each healthcare professional’s demographic attributes, values, and empathetic capacity intersected to convey positive or adverse effects (Zhang et al., 2018). Since then, gender has been found to influence nurses’ health *and* professional quality of life, suggesting inter-role conflict as a potential contributor (Ruiz-Fernandez et al., 2020a; 2020b).

The nursing and midwifery workforce remains female-dominated globally resulting in tension imposed by conflicting work and family responsibilities (Buchan et al., 2022; Zurlo et al., 2020). Inter-role conflict occurs when ‘expectations and behaviours associated with one role are not consistent with the expectations and behaviours associated with another’ (American Psychology Association [APA], Online Dictionary, 2024). Female nurses have reported higher levels of work-family conflict, anxiety, and depression, with many choosing to work night duty to help balance their family commitments (Zurlo et al., 2020).

Personal attributes of temperament, caring ability, and resilience also convey varying effects, especially when overlaid by organisational influences such as acuity, throughput, nurse: patient ratios, and skill mix (Barr, 2022; Cankaya and Dikmen, 2020; Peng et al., 2021; Ruiz-Fernandez et al., 2020a). Intrinsic and extrinsic variables alter the safety climate in which nurses and midwives work, affecting care quality and professional power (Cankaya and Dikmen, 2020; Peng et al., 2021). For instance, Ruiz-Fernandez et al. (2020a) detected higher levels of CS among hospital employees compared to primary care professionals. Peng and colleagues (2021) discovered more than half (52.1%) their sample of Chinese registered nurses had experienced workplace bullying, the effects of which were moderated by their resilience levels.

Exposure to work-related traumatic events and post-traumatic stress further influences nurses’ and midwives’ professional quality of life (Ruiz-Fernandez et al., 2021). Consequently, clinicians in acute care environments, such as emergency departments and intensive care units, may be at increased risk of adverse health outcomes (Ruiz-Fernandez et al., 2021). A quantitative cross-sectional study of 86 Australian emergency department nurses using Stamm’s (2010) Professional Quality of Life Scale (ProQOL, v.5) and open-ended questions revealed moderate to high CS and moderate to low CF. Several work-related sources of stress, such as patient mix, contributed (O’Callaghan et al., 2020).

Education and social support can be protective. Possessing a post-graduate certificate or diploma predicts work satisfaction (O’Callaghan et al., 2020). Social support ameliorates work-related stress for nurses and midwives employed in acute care environments, especially for women (Ruiz-Fernandez et al., 2021; Zurlo et al., 2020). Therefore, the physical and psychological health of nurses and midwives working in high-pressure environments and their ability to access social support and ongoing professional development is important and worthy of attention (O’Callaghan et al., 2020; Ruiz-Fernandez et al., 2021; Zurlo et al., 2020).

Empirical research findings underscore the importance of identifying how optimal professional quality of life is achieved and maintained. Doing so augments physical and psychological well-being and maximises organisational outcomes such as health workforce retention (Adelson et al., 2021). As the Coronavirus disease 2019 (COVID-19) pandemic politicised healthcare and the role of front-line workers, forcing many to consider leaving the profession, this research remains vital (Adelson et al., 2021).

## Background/rationale

Workforce figures reveal that while 372, 759 nurses and midwives are registered with the Australian Health Practitioner Regulation Agency, the annual growth rate of 1.7% registered in 2022 was the lowest of all Australian health professionals (Australian Institute of Health & Welfare [AIHW], 2024b). Approximately 5300 (1.4%) identify neonatal care as their area of clinical speciality (Australian Government Department of Health, 2020). Neonatal nurses work full-time, part-time, or casually and are defined as:

Registered nurses or registered midwives who work primarily with neonates and their families in a neonatal care facility. Roles may include any combination of direct clinical care, management, education, or research specific to neonates, their families and/or the neonatal care environment (Australian College of Neonatal Nurses [ACNN], 2019, p. 7).

Demand for technologically advanced neonatal care in Australia remains steady (AIHW, 2024a). Of the 315, 705 Australian births recorded in 2021, 8.2% of babies were born between 20 and 36 weeks’ gestation (AIHW, 2024a). In 2021, almost one-fifth (17%) of all Australian live-born babies, up from 15.9% in 2010, were admitted to either a special care nursery (SCN) or neonatal intensive care unit (NICU) at birth (AIHW, 2024a). NICUs are equipped and staffed to deliver highly specialised acute care, whereas SCNs are resourced to provide sub-acute care (Australasian Health Infrastructure Alliance [AHIA], 2019).

Maternity and neonatal services are delineated and funded across six levels based on frameworks such as the Maternity and Neonatal Service Capability Policy GL2022_002 (NSW Ministry of Health, 2022). These levels reflect the activity and clinical complexity a facility can provide safely (NSW Ministry of Health, 2024). Admission criteria for babies entering a SCN or NICU adhere to strict gestational age and birthweight guidelines. They are also dictated by factors such as babies’ requirements for assisted ventilation, cardiorespiratory monitoring, intravenous fluids, and other medical or surgical interventions (Chow et al., 2024). Providing this time-sensitive and technologically advanced care to critically ill babies is morally and ethically challenging for neonatal clinicians (Green et al., 2016; Welty, 2019).

An imperative to practice according to ethical principles of beneficence, non-maleficence, autonomy, and justice is paramount (Haahr et al., 2020). Nurses’ inability to deliver quality care in accordance with their professional values causes ethical distress (Haahr et al., 2020). As one salient example, organisational infection prevention and control decisions made in response to the COVID-19 pandemic limited neonatal workforce availability and restricted parental access to neonatal units (Shaw et al., 2021). Parental presence and involvement in care significantly increase parent-infant bonding (Kutahyalioglu and Scafide, 2022). Yet, policy decisions undermined family-centred, neurodevelopmental care philosophies integral to contemporary neonatal care and severely compromised neonatal nurses’ professional identities (Shaw et al., 2021).

A recently published literature review affirmed that neonatal clinicians of advanced age and experience were not immune to work-related stress (Lowe et al., 2022). However, they could remain satisfied with their work while experiencing these negative feelings (Lowe et al., 2022). Supportive clinical environments with strong safety cultures and high-level inter-professional collegiality enriched neonatal nurses’ mental and physical health, greatly enhancing staff well-being and retention (Lowe et al., 2022). Renewed stakeholder commitment to improving neonatal clinicians’ health and well-being and additional research into structural factors, such as skill mix and patient acuity, affecting neonatal nurses’ professional quality of life were requested (Lowe et al., 2022).

Based on integrative review findings, a Mixed Methods Grounded Theory study was commenced to explain Australian neonatal nurses’ professional quality of life. This two-phase project is guided by a Pragmatic theoretical perspective and an explanatory sequential design (quant->QUAL) (Draucker et al., 2020).

## Gap in the literature

Managing COVID-19 pandemic responses imposed an unprecedented strain on the international nursing and midwifery workforce, illuminated healthcare system fragilities, and exacerbated existing global nursing shortages (Buchan et al., 2022). However, a lack of research has been conducted on Australian clinicians’ well-being, especially those working in relatively small nursing sub-specialities such as neonatal care.

## Aim

The study aimed to establish the demographic profile of surveyed Australian neonatal nurses, their self-reported professional quality of life status, and the strength and direction of relationships between these variables.

## Methods

### Study design

This was a descriptive, cross-sectional study. An online survey was developed using the Qualtrics^TM^ platform and distributed via email between 15 December 2021 and 31 January 2022. Due to a low response rate, we extended the study for 6 weeks and issued three social media prompts (via Twitter and LinkedIn).

### Ethics approval and recruitment

University of Technology Sydney Human Research Ethics Committee (ETH21-6124) provided ethical approval. ACNN Executive Group permission was obtained to contact their general members (*n* = 950) and invite them to complete an online survey. All Australian registered nurses and midwives who, by their ACNN membership, stipulated neonatal care as their clinical speciality area were eligible to participate in this study. All respondents provided informed consent before participating.

### Variables and measurements

Demographic and professional information about age, gender, employment status, current role, highest education qualification, and years of experience in neonatal care were collected. All participants were asked to complete the ProQOL-21 scale (Heritage et al., 2018). The ProQOL-21 scale is a 21-item, two-armed screening scale measuring CS and CF developed by Heritage and colleagues (2018) in response to concerns about the construct validity of Stamm’s (2010) original- and widely used- three-armed, 30-item scale (Pro-QOL, v.5). The revised scale was developed following a Rasch analysis of responses from 1615 Australian registered nurses.

All negatively worded items and those with a limited shared variance were removed. Some questions’ neighbouring item response categories were collapsed to further aid clarity and administration efficiency. Before survey distribution, coding modifications were made reflecting collapsed response categories recommended by Heritage and colleagues (2018, p. 8). Participants ranked their ‘positive and negative’ professional ‘helping’ experiences within the last 30 days on a five-point Likert scale from 1 = Never to 5 = Very Often (Heritage et al., 2018). The ProQOL-21 scale demonstrated excellent construct validity (Heritage et al., 2018, p. 14).

### Statistical analysis

Data were analysed using SPSS (v.27). Internal consistency was estimated using Cronbach’s alpha. Statistical significance was determined using a *p*-value of 0.05. Non-statistically significant Kolmogorov–Smirnov and Shapiro–Wilk Tests indicated normally distributed outcome variables (Field, 2018). Total CS scores were calculated by summing responses to questions 1, 2, 7, 10, 11, 13, 15, 17, 20, and 21. Questions 3, 4, 5, 6, 8, 9, 12, 14, 16, 18, and 19 were added to calculate total CF scores. Raw scores (z scores) *were not* converted to t-scores because the sample size was greater than 30 and indicated a normal distribution (CS: W = 0.959, *p* = .069; CF: W = 0.964; *p* = 0.114). Internal consistency of the ProQOL-21 scale in this study was checked using Cronbach’s alpha with α = 0.927 for CS (excellent) and α = 0.807 for CF (good) (Field, 2018).

CS and CF scores were appraised for skewness and kurtosis. The CS scores were negatively, and the CF scores were positively skewed. Both CS and CF scores were platykurtic (Field, 2018). Due to skewed distribution and ordinal data, central tendency was reported using the median and interquartile range (Field, 2018). Spearman rank-order correlation coefficient determined ordinal variables’ association significance. Accepted conventions [0.00–0.19 = very weak, 0.20–0.39 = weak, 0.40–0.59 = moderate, 0.60–0.79 = strong, 0.80–1.0 = very strong] were used to determine the strength, direction, and statistical significance (Field, 2018). All assumptions were met.

## Results

### Participants

Our final sample consisted of 53 registered nurses after removing three incomplete surveys containing only demographic information. All participants identified as women. Only eight percent (*n* = 4) were casually employed; the rest worked in permanent part-time (*n* = 23, 43%) or full-time (*n* = 26, 49%) positions. Over 70% of our sample worked in clinical settings (*n* = 38, 71.7%), either in a SCN (*n* = 8, 15.1%), NICU (*n* = 9, 17%), or both (*n* = 21, 39.6%). The remainder had roles in education, management, or research. Most respondents (*n* = 47, 88.7%) were over 30 years of age and had a graduate diploma or higher qualification (*n* = 33, 62.3%). Over one-third of respondents had a Master’s degree (*n* = 18, 34%) or doctorate (*n* = 2, 3.8%). This cohort was very experienced. Almost half had over 20 years of clinical experience in neonatal care (*n* = 25, 47.2%) (see Table 1 Australian neonatal nurse demographic profile).

**Table 1.** Australian neonatal nurse demographic profile.

Age (*n*/%)	
20–30 years	6 (11.3%)
31–40 years	11 (20.8%)
41–50 years	11 (20.8%)
51–60 years	12 (22.6%)
Over 60 years	13 (24.5%)
Gender	
Female	53 (100%)
Current employment status	
Casual	4 (8%)
Permanent part-time	23 (43%)
Permanent full time	26 (49%)
Current role	
Clinical-special care only (SCN)	8 (15.1%)
Clinical-intensive care only (NICU)	9 (17%)
Clinical both SCN and NICU	21 (39.6%)
Education	11 (20.8%)
Management	1 (1.9%)
Research	3 (5.7%)
Highest qualification	
Certificate	5 (9.4%)
Bachelor’s degree	15 (28.3%)
Graduate dip/Certificate	13 (24.5%)
Master’s degree	18 (34%)
Doctorate	2 (3.8%)
Years of experience in neonatal care	
0–5 years	7 (13.2%)
6–10 years	8 (15.1%)
11–15 years	7 (13.2%)
16–20 years	6 (11.3%)
>20 years	25 (47.2%)

## Outcome data

### Main results

The median CS score was 28 (IQR = 8.50), and the CF score was 25 (IQR = 7.00). Compared to the 25th, 50th, and 75th percentile cut-off points used to indicate a low, medium, or high-level screening in the ProQOL-21 scale, about one-fifth (18.85%) of nurses reported low-level, 30.25% medium-level, and 50.9% high-level CS. No scores indicated a low-level CF. Instead, 15.1% stated moderate-level, and 84.9% of scores exceeded the cut-off point, suggesting a high-level screening (Heritage et al., 2018, p. 10). While 39.6% of scores exceeded the median score of 28 for CS, 47.2% exceeded the median of 25 for CF, indicating respondents felt more fatigued than satisfied (See Supplemental material).

### Correlations

Spearman’s correlation (*r*) measured the strength and direction of relationships between the ordinal variables. Field (2018) recommends Spearman’s correlation as a suitable choice for small data sets demonstrating monotonicity, sets of paired ordinal variables, and tiered ranks. One weak but statistically significant positive correlation was detected between years of experience in neonatal care and CS (r = 0.277, 95% CI [0.002, 0.513]) (see Table 2 Spearman’s Correlations).

**Table 2.** Spearman’s correlations.

	Age	Employment status	Current role	Highest qualification	Years experience
Total CS score	r = .260	r = .014	r = .106	r = −.024	r = .277
	95% CI	95% CI	95% CI	95%CI	95% CI
	[−.015, .499]	[−.257, .283]	[−.170, .367]	[−.293, .248]	[.002, .513]
Total CF score	r = −.087	r = −.108	r = .120	r = .148	r = −.083
	95% CI	95% CI	95% CI	95% CI	95% CI
	[−.350, .188]	[−.368, .168]	[−.156, .379]	[−.129, .404]	[−.346, .192]

*Correlation is significant at the 0.05 level (2-tailed). CI 95% (2-tailed).

### Scores exceeding the median for CS and CF

Participant percentages within each demographic sub-band with a CS or CF score exceeding the sample median were calculated. A linear trend between age and CS revealed that staff became more professionally satisfied as they aged. However, this may have more to do with the fact that nurses in non-clinical roles such as management and research were more satisfied than those in clinical positions. Casually employed staff were more satisfied than those in permanent full-time or part-time roles.

Nurses who rotated between special care nurseries and neonatal intensive care units were more satisfied than clinicians employed exclusively in either area. Neonatal nurse educators were least satisfied in their current roles. Counterintuitive trends between education and satisfaction were observed, with less qualified staff reporting greater satisfaction than their highly educated colleagues. The influence of neonatal experience on CS revealed two distinct satisfaction waves. Mid (6–10 years experience) and late-career nurses (with over 20 years experience) reported greater satisfaction than either early (0–5 years experience) or mid/late-career (11–20 years experience) nurses.

Nurses aged 31–40 or over 50 reported higher CF scores than those aged under 30 and between 41 and 50 years. Permanent part-time employees reported more fatigue than nurses in permanent full-time or casual positions. Neonatal nurse educators revealed notably high CF rates. Nurses employed exclusively in special care environments returned proportionally higher CF scores than those who rotated between clinical areas or worked solely in neonatal intensive care. Nurses working in research roles recorded the lowest CF scores. Nurses with higher degree qualifications and over 16 years of clinical experience revealed higher CF scores than less educated, less experienced nurses. Neonatal nurses with 6–10 years of neonatal experience reported lowest CF rates (See Supplemental material).

## Discussion

This quantitative study aimed to capture a cross-sectional view of Australian neonatal nurses’ demographic profiles and self-reported professional quality of life. It also aimed to determine the strength and direction of relationships between nurses’ age, employment status, current role, highest education qualification, years of experience in neonatal care, and their reported CS and CF scores. Surveyed Australian neonatal nurses revealed moderate to high-level CS and high CF. Nurses in clinical roles reported higher CF scores than those in non-clinical roles. Extrapolating those findings, implies inter-role conflict, social support, moral distress, and increased acuity-adjusted workload as areas of concern.

### Incidence rates

Zhang and colleagues (2018) conducted a meta-analysis of epidemiological studies relating to CS, CF, and burnout among almost 8000 nurses that revealed prevalence rates of 48% (CS), 53% (CF), and 54% (burnout), respectively. Analysis showed that while rates were not significantly associated with age, experience level, and gender, registered nurse status and higher education effectively countered CF.

While our sample appeared to grow more satisfied with age, we observed that education and experience did not guard against CF. Notwithstanding that capacity for co-existing satisfaction and fatigue has long been recognised, providing nurses with work environments that optimally engender positive feelings benefits organisations, staff, and patients (Sacco and Copel, 2017). Because retaining a robust workforce is crucial to ensuring an effective health system, identifying strategies to bolster a sense of professional satisfaction is vital to prevent nurses from losing hope and permanently leaving the profession (Peters, 2018).

This cross-sectional study was conducted at the end of 2021, following almost 2 years of a global health emergency initiated by the COVID-19 pandemic. Research conducted during this time revealed that some nurses and midwives had expressed plans to resign because of genuine safety concerns, inadequate staffing, and high patient acuity (Adelson et al., 2021; Halcomb et al., 2020). Buchen et al. (2022) foresee a pandemic-related vicious cycle in which demand for nurses exceeds supply, fuelling absenteeism and ongoing attrition. Strategies such as resilience training, clinical supervision, and reflective practice reportedly counter these effects by engendering a sense of personal agency (Peters, 2018; Sundgren et al., 2020).

Neonatal nurses reported feeling afraid, underprepared, and constrained by imposed structural barriers preventing the provision of holistic, family-centred care central to their professional philosophies (Shaw et al., 2021). While direct exposure to COVID-19-positive patients may have been less common in maternity and neonatal settings than in other clinical speciality areas, requirements for social distancing, rapid antigen testing, and isolation led to reports of poor interprofessional collegiality, moral distress, reduced safety and care quality and inter-role conflict (Haidari et al., 2021). Furthermore, some work-based stress and satisfaction sources may be gender specific.

### Inter-role conflict and social support

The global nursing community remains female-dominated (Buchan et al., 2022). Our sample all identified as women. Those aged 31–40 years and over 50 revealed the highest CF scores. Their reports of sub-optimal satisfaction and increased fatigue coincided with ages when women experience life-changing events such as childbirth and menopause and are committed to caring responsibilities outside the workplace (AIHW, 2024a). Female nurses’ psychological health is disproportionately affected by the pressures of balancing work and family commitments (Zurlo et al., 2020). Therefore, viewing structural barriers impacting this predominantly female workforce’s professional quality of life is essential.

It has been over a decade since Green, (2012, p. 1) stated, ‘the implications for care are embedded in the personal and social values and experiences associated with gender, power, and politics’ and petitioned for a moral voice valuing autonomy and professional relationships. Zhu et al. (2021) maintain that nurses’ emotional states and self-identity continue to be eroded by gender biases evident within the health system’s hierarchal structures and that their health and well-being can be improved through gender-specific interventions, such as social support for women.

Neonatal nurse educators and permanent part-time employees reported higher CF scores than neonatal nurses with differing roles or employment statuses. These findings suggest that societal and hierarchical constraints affect female nurses’ professional quality of life. These observations imply that limited self-determination capacity may provoke inter-role conflict and that neonatal nurses with little flexibility concerning their work assignments may be unable to access the social support required to offset work-related stress.

### Moral distress

While Australian mid-career nurses with 6-10 years of neonatal experience reported the lowest CF scores, more highly educated and experienced clinicians reported feeling more fatigued than their less educated and experienced peers. Over the last three decades, empirical research and technological and pharmacological advances have progressively reduced viability limitations while expanding the range of acceptable interventions for critically ill babies (Welty, 2019).

Moral distress arises when clinicians are organisationally constrained from doing what they know is correct or, having decided to do so, do not follow through with a morally sound decision. In some circumstances, neonatal nurses may feel unable to act per their ethical and moral convictions or may believe that their professional decision-making capacity has been restricted by forces beyond their control (Haahr et al., 2020). When evaluating compromised, untruthful, and futile care concerning neonatal nurses’ moral distress and burnout, Barr (2022) found that compromised care led to demoralisation and exhaustion; untruthful care was demoralising, and futile care predicted exhaustion (Barr, 2022).

Nurses’ moral distress is exacerbated by injustice, conflicting responsibilities, perceived advocacy capacity, team dynamics, and rostering challenges (Kanaris, 2023; Vittner et al., 2021). Nurses with professional insight and an appreciation of the ramifications of action and inaction are more likely to be affected. Encouraging nurses to reflect ethically and develop emotional regulation and self-care strategies within work environments that value team psychological safety reduces moral distress and improves patient safety (Kanaris, 2023; Morley et al., 2022). Methods to reduce moral distress, such as transformational leadership, interprofessional collaboration, information sharing, and procedural competence, enhance neonatal nurses’ decision-making capabilities and capacity to provide holistic, family-centred care (Barr, 2022; Kanaris, 2023; Vittner et al., 2021).

### Staffing and acuity-adjusted workload

Clinicians employed exclusively in SCN environments returned higher CF scores than those rotating through all acuity levels or working solely in neonatal intensive care. These findings insinuate that global funding deficits and trends toward rising acuity-adjusted workload contributing to missed care are now becoming a safety and quality concern for Australian neonatal nurses. Missed care elements such as anticipatory parental guidance, providing breastfeeding support, and coordinating care, jeopardise the attainment of longer-term organisational and population outcomes, extending a baby’s length of hospital stay and negatively impacting their long-term growth and development (Lake et al., 2020). Because nurses are not oblivious to these implications, the burden of being unable to provide necessary care may undermine their professional quality of life (Lake et al., 2020).

Perhaps due to COVID-19-related staffing challenges directly impacting clinical staff our study findings also highlighted how nurses in non-clinical roles enjoyed greater professional satisfaction than those in clinical positions. It seems clear that new methods of determining safe staffing levels are required to enhance neonatal and family health outcomes and improve neonatal nurses’ professional quality of life. Professional judgement is relied upon without an evidence-based tool to balance patient acuity with anticipated throughput and available skill set (Griffiths et al., 2020). Following the COVID-19 pandemic, scant progress has been made concerning safe staffing in acute care environments. However, the Lancet Global Health Commission on Primary Health Care has called for key stakeholders to collaboratively develop tools and strategies to finance efficient healthcare delivery (Hanson et al., 2022).

The National Association of Neonatal Nurses [NANN] (2021) also noted the need for safe and appropriate staffing in neonatal care environments. The rationale considered newborn babies’ requirements for constant vigilance and emergency, routine, and palliative care. Consequently, NANN recommended: (1) staffing ratios based on front-line nurses’ assessment of each infant’s physiological and psychosocial acuity, (2) matching infants’ care requirements with staff skill mix, (3) collaborative decision making, (4) quality improvement strategies to evaluate and minimise missed care, (5) judicious allocation of human and material resources to maximise the use of healthcare funding, and (6) census appraisal to accommodate seasonal fluctuations (NANN, 2021).

Our findings suggest that the stress imposed on nurses in hybrid roles, like clinical education, and rapid throughput in high-acuity areas, such as special care nurseries, may have been underappreciated. In both instances, the ability to meet key performance indicators may be undermined by poor skill mix, low medical support, and sub-optimal nurse-to-patient ratios. Babies with high-acuity needs continue to be cared for in special care nurseries classified as delivering low-dependency care that may be contributing to chronic underfunding (AHIA, 2019; Hanson et al., 2022; NSW Ministry of Health, 2024). Consequently, special care nurseries’ designation, design, and funding must be continuously reappraised.

## Limitations

This study was the first to gather data from a national sample of Australian neonatal nurses’ regarding their professional quality of life, receiving a 5.58% response rate. While low response rates to studies surveying members of professional nursing organisations are not unprecedented (Beck et al., 2017), this survey was sent to professional nursing organisation members after 2 years, during which the COVID-19 pandemic severely disrupted neonatal care provision (Shaw et al., 2021). Consequently, some potential participants may have felt physically depleted and disinclined to participate. The small sample size and self-report nature of the survey responses were distinct limitations; therefore, no claim of generalisability beyond the surveyed sample can be made. However, responses were received in each category and demographic band, revealing interesting demographic trends and interrelationships between variables. These initial results are informative and noteworthy. The weak but statistically significant positive correlation between years of experience in neonatal care and CS warrants further investigation.

Sampling, self-selection, response, and recall bias are common concerns relating to cross-sectional survey designs, especially when the sample is non-randomised and not representative. The potential for inaccurately or incompletely answered questions also exists (Popovic and Huecker, 2023). In this instance, the risk of sampling bias was minimised by circulating the survey and reminders to all ACNN members and using an abridged and psychometrically enhanced survey to collect anonymous responses and encourage a high participation rate (Heritage et al., 2018).

When comparing a range of sampling, recruitment, and data collection options used in multidisciplinary healthcare studies, Hutchinson and Sutherland (2019) concluded that emailed surveys and reminders optimally balanced response rates with research cost and effort. In this case, recall bias associated with retrospective surveys was minimised by the tool’s short duration and requirements for participants to report their recent experiences (Heritage et al., 2018; Popovic and Huecker, 2023). Results were reported regardless of their statistical significance, thus reducing the risk of publication bias (Baldwin et al., 2022; Popovic and Huecker, 2023).

Free-text responses about previously reported contributors to nurses’ professional quality of life, such as empathy, resilience, and safety culture, were not sought but were explored during focus groups and interviews in this study’s subsequent qualitative phase (Charmaz, 2014).

## Implications for nursing and midwifery practice

Identifying organisational strategies to enhance the professional quality of life of Australia’s female-dominated neonatal workforce is urgently needed. Systematic changes that enable a sense of personal agency are necessary to counter gender biases and structural barriers impeding neonatal nurses’ professional quality of life. Facilitating such improvements would increase workplace safety and reduce clinicians’ moral distress when constrained by system deficiencies beyond their control. Doing so may inflate neonatal nurses’ self-determination capacity and professional worth, strengthening their commitment to the profession and aiding workforce retention.

Such refinements would undoubtedly amplify nurses’ ability to provide evidence-based care to vulnerable newborns and their families, enhancing their health outcomes and improving neonatal nurses’ professional quality of life. In time, these quality improvements may also contribute to policy and practice changes benefitting the well-being and retention of the broader nursing and midwifery populations.

## Conclusion

This descriptive cross-sectional study was the first to gather data from a national sample of neonatal nurses regarding their professional quality of life. Our findings defied the belief that education and experience safeguard neonatal nurses against work-related stress. Furthermore, they emphasised that strategies are urgently needed to offset the fatigue reported by SCN staff, neonatal nurse educators, and nurses with caring responsibilities outside the workplace. These results support previously published findings indicating inter-role conflict, social support, moral distress, staffing, and acuity-adjusted workload concern neonatal nurses and midwives.

Alterations to neonatal service delineation, funding, and how the staff is recruited, professionally developed, and retained must be considered. Broad-scale policy reform and flexible staffing arrangements are required to combat pressure exerted by increased acuity-adjusted workload evident in neonatal units. The long-lasting effects of the COVID-19 pandemic on the neonatal nurse workforce and the relevance of these findings for other healthcare professions also warrant further exploration.

## Supplemental Material

Supplemental Material - Australian neonatal nurses’ professional quality of life: A descriptive cross-sectional study Supplemental Material for Australian neonatal nurses’ professional quality of life: A descriptive cross-sectional study by Patricia L. Lowe, Samantha Jakimowicz, Tracy L. Levett-Jones and Daniel Demant in Journal of Child Health Care.
