# Supplementary material for: Australian neonatal nurses’ professional quality of life: A descriptive cross-sectional study
Source: J Child Health Care. 2025 May 2;30(2):245–59. doi: 10.1177/13674935251339351 (PMC13168593; doi:10.1177/13674935251339351)
Supplement: Supplemental Material - Australian neonatal nurses’ professional quality of life: A descriptive cross-sectional study [file sj-pdf-1-chc-10.1177_13674935251339351.pdf]

Supplementary material: Percentile cut-points and CS/CF scores per demographic band exceeding the median score.

**Percentile cut points**	25th (LOW) =21	50th (MED) =26	75th (HIGH) =30	MEDIAN
**Compassion satisfaction (CS)**	18.85%	30.25%	50.9%	= 28<br>39.6% exceeded the median
**Compassion fatigue (CF)**	=16<br>0%	=20<br>15.1%	=25<br>84.9%	= 25<br>47.2% exceeded the median

**Demographic band/number**	**Response range CS**	**n/% exceeding the median (28) for CS**	**Response range CF**	**n/% exceeding the median (25) for CF**
**Age**				
20-30 (n=6)	14-28	1 (16.7%)	18-38	3(50.0%)
31-40 (n=11)	17-34	4 (36.4%)	22-35	7(63.6%)
41-50 (n=11)	22-36	6 (54.5%)	18-35	5(45.5%)
51-60 (n=12)	14-36	6 (50.0%)	18-35	8(66.7%)
>60 (n=13)	15-35	9 (69.2%)	18-32	8(61.5%)
Total=53	14-36	-	18-38	-
**Employment status**				
Casual (n=4)	25- 36	3 (75.0%)	22-35	1(25.0%)
PPT (=23)	14-35	9 (39.1%)	18-38	17(73.9%)

PFT (n=26)	14-34	16 (61.5%)	18-36	13(50.0%)
Total= 53	14-36	-	18-38	-
**Current role**				
SCN (n=8)	14-36	4(50.0%)	18-35	5(62.5%)
NICU (n=9)	14-34	4(44.4%)	18-36	4(44.4%)
SCN & NICU (n=21)	16-36	12(57.1%)	18-38	11(52.4%)
Education (n=11)	18-32	5(45.4%)	20-32	10(90.9%)
Management (n=1)	28	1(100.0%)	23	0 (0.0%)
Research (n=3)	27-34	2(66.7%)	18-29	1(33.3%)
Total=53	14-36	-	18-38	-
**Highest education qualification**				
Certificate (n=5)	14-29	3 (60.0%)	19-35	3(60.0%)
Bachelor’s degree (n=15)	14-35	9(60.0%)	18-36	7(46.7%)
Grad cert/Dip (n=13)	16-36	7(53.8%)	18-38	7(53.8%)
Master’s degree (n=18)	15-36	8(44.4%)	19-35	12(66.7%)
Doctorate (n=2)	22-27	0 (0.0%)	29-32	2(100%)
Total= 53	14-36		18-38	-
**Years neonatal experience**				
0-5 years (n=7)	14-27	1(14.3%)	24-36	4(57.1%)
6-10 years (n=8)	18-33	5(62.5%)	18-38	4(50.0%)
11-15 years (n=7)	14-32	3(42.9%)	20-35	4(57.1%)
16-20 years (n=6)	20-32	3(50.0%)	21-32	5(83.3%)
>20 years (n=25)	15-36	15(60.0%)	18-35	14(56.0%)
Total= 53	14-36	-	18-38	-
